# Rapid evolution of mitochondrion-related genes in haplodiploid arthropods

**DOI:** 10.1186/s12915-024-02027-4

**Published:** 2024-10-10

**Authors:** Yiyuan Li, Gregg W. C. Thomas, Stephen Richards, Robert M. Waterhouse, Xin Zhou, Michael E. Pfrender

**Affiliations:** 1 State Key Laboratory for Managing Biotic and Chemical Threats to the Quality and Safety of Agro-Products, Key Laboratory of Biotechnology in Plant Protection of Ministry of Agriculture and Rural Affairs, Key Laboratory of Green Plant Protection of Zhejiang Province, Institute of Plant Virology, Ningbo University, Ningbo, 315211 China; 2 Department of Biology, Indiana University, Bloomington, IN USA; 3 Department of Computer Science, Indiana University, Bloomington, IN USA; 4 Current Address: Informatics Group, Harvard University, Cambridge, MA USA; 5 Human Genome Sequencing Center, Department of Human and Molecular Genetics, Baylor College of Medicine, One Baylor Plaza, Houston, TX USA; 6 Department of Ecology & Evolution and Swiss Institute of Bioinformatics, University of Lausanne, 1015 Lausanne, Switzerland; 7 Department of Entomology, College of Plant Protection, China Agricultural University, Beijing, 100193 China; 8 Department of Biological Sciences, University of Notre Dame, Notre Dame, IN USA; 9 Environmental Change Initiative, Notre Dame, IN USA

**Keywords:** Gene family evolution, Evolutionary rate, Gene duplication, Oxidative phosphorylation genes, Hymenoptera, Thysanoptera, Phthiraptera, Mesostigmata, Trombidiformes

## Abstract

**Background:**

Mitochondrial genes and nuclear genes cooperate closely to maintain the functions of mitochondria, especially in the oxidative phosphorylation (OXPHOS) pathway. However, mitochondrial genes among arthropod lineages have dramatic evolutionary rate differences. Haplodiploid arthropods often show fast-evolving mitochondrial genes. One hypothesis predicts that the small effective population size of haplodiploid species could enhance the effect of genetic drift leading to higher substitution rates in mitochondrial and nuclear genes. Alternatively, positive selection or compensatory changes in nuclear OXPHOS genes could lead to the fast-evolving mitochondrial genes. However, due to the limited number of arthropod genomes, the rates of evolution for nuclear genes in haplodiploid species, besides hymenopterans, are largely unknown. To test these hypotheses, we used data from 76 arthropod genomes, including 5 independently evolved haplodiploid lineages, to estimate the evolutionary rates and patterns of gene family turnover of mitochondrial and nuclear genes.

**Results:**

We show that five haplodiploid lineages tested here have fast-evolving mitochondrial genes and fast-evolving nuclear genes related to mitochondrial functions, while nuclear genes not related to mitochondrion showed no significant evolutionary rate differences. Among hymenopterans, bees and ants show faster rates of molecular evolution in mitochondrial genes and mitochondrion-related nuclear genes than sawflies and wasps. With genome data, we also find gene family expansions and contractions in mitochondrion-related genes of bees and ants.

**Conclusions:**

Our results reject the small population size hypothesis in haplodiploid species. A combination of positive selection and compensatory changes could lead to the observed patterns in haplodiploid species. The elevated evolutionary rates in OXPHOS complex 2 genes of bees and ants suggest a unique evolutionary history of social hymenopterans.

**Supplementary Information:**

The online version contains supplementary material available at 10.1186/s12915-024-02027-4.

## Background

Eukaryotes have repeatedly acquired intimate symbiotic microorganisms, including mitochondria, plastids, and microbiome, which provide great benefits to the eukaryote hosts, facilitating adaptation and diversification of the hosts [1, 2]. The most ancient symbionts, mitochondria, have formed obligate interdependent relationships with their eukaryote hosts. Mitochondrial genes and nuclear genes cooperate closely with each other in oxidative phosphorylation, as well as maintaining mitochondrial replication, transcription, and translation [3]. Mitochondrial genomes in animals have higher mutation rates and lower efficacy of selection than nuclear genomes [4–7]. As a result, mitochondrial genomes often have faster molecular evolutionary rates than nuclear genomes [8–10]. These evolutionary rate differences can generate inter-genomic incompatibilities that have been implicated in a variety of contexts including sexual selection, hybrid breakdown, and speciation [11]. Understanding these inter-genomic interactions is a key to addressing fundamental questions in evolutionary biology, such as the evolution of gene–gene interactions, and the processes of adaptation and speciation [12–16].

Despite the functional importance of mito-nuclear interactions, mitochondrial and nuclear genes have shown dramatically different evolutionary rates among arthropods [17–19]. Species in the order Hymenoptera (sawflies, wasps, bees, and ants) have elevated evolutionary rates (amino acid substitutions per site per million years) in both mitochondrial and mitochondrion-related nuclear genes, but not for the single-copy nuclear genes, compared to other arthropod species [19–21].

Different factors could influence the evolutionary rate of mitochondrial genes, including genetic drift, selection, and mutation rate [3]. Multiple hypotheses have been proposed to explain the elevated rate of molecular evolution in hymenopterans. First, the vast majority of hymenopterans have haplodiploid sex-determination systems [22]. Many of them also have parasitic lifestyles and varying degrees of eusociality. These unique features could lead to repeated founder effects and a small effective population size, which can increase the influence of genetic drift leading to the accumulation of slightly deleterious mutations and elevated evolutionary rates in the mitochondrial and nuclear genomes [23–25]. However, the observed similar evolutionary rate of single-copy nuclear genes between hymenopterans and other insects contradicts this hypothesis. Alternatively, positive selection on the oxidative phosphorylation (OXPHOS) pathway could lead to the observed rapid evolution in both mitochondrial and nuclear OXPHOS genes [18, 20, 26]. In addition, the accumulation of deleterious mutations in mitochondrial genes could drive compensatory changes in related nuclear genes. The fixation of beneficial mutations in nuclear genes could, in turn, reduce the cost of subsequent deleterious mutations in the mitochondrial genome promoting the fixation of additional mitochondrial mutations [27, 28]. As previous studies often focus on hymenopterans, the role of haplodiploidy in the fast evolution of mitochondrial genes is not clear. In addition, previous studies [18, 19, 21] were based on the transcriptome dataset from Misof et al. [29] or conserved nuclear genes [20, 28]. In these studies, only the evolutionary rate of extremely conserved single-copy genes was tested. Therefore, it is also not clear whether less conserved nuclear genes in the genomes would show the same or different evolutionary patterns.

To test these hypotheses in other haplodiploid lineages, we use an extensive arthropod whole genome dataset of 76 arthropod species from 17 orders spanning 500 million years of evolution [30–32]. This dataset includes five independent origins of haplodiploidy [33] and two different types of haplodiploid systems, arrhenotoky and parental genome elimination (PGE). In arrhenotoky species, unfertilized eggs develop into male individuals, whereas PGE males develop from fertilized eggs but then the paternal chromosomes are removed or not passed to offspring [22, 33, 34]. Our dataset includes two independent PGE lineages, the western predatory mite (*Metaseiulus occidentalis*) [35–37] and the body louse (*Pediculus humanus*) [38], and three independent arrhenotoky lineages, the two-spotted spider mite (*Tetranychus urticae*) [39], the western flower thrips (*Frankliniella occidentalis*) [34], and hymenopterans. The inclusion of multiple independent evolutionary origins and different types of haplodiploidy greatly enhances our ability to describe the overall molecular evolutionary patterns of mitochondrial and nuclear genes in haplodiploid species. In addition, as we are using complete genome data, we can study not only single-copy nuclear genes but also 5746 orthologous nuclear genes that are found in most of the sequenced arthropod genomes. Based on this genome dataset, we investigate the evolutionary rate of mitochondrial genes and nuclear genes of haplodiploid arthropod species. We show that the five lineages of haplodiploid species all have fast-evolving genes related to the mitochondrion but not for genes unrelated to the mitochondrion. As a small population size would affect both mitochondrion-related and non-related nuclear genes, this finding rejects the hypothesis that the small effective population size of haplodiploid species increases the influence of genetic drift, leading to the fast-evolving mitochondrion-related genes. In Hymenoptera, bees and ants have even faster evolutionary rates in mitochondrion-related genes than basal hymenopterans, including sawflies and wasps, suggesting other processes could affect the evolutionary pattern. Lastly, haplodiploid species as well as bees and ants have unique gene family expansions and contractions that could imply functional differences in these groups.

## Results

### Data acquisition

**Fig. 1:**
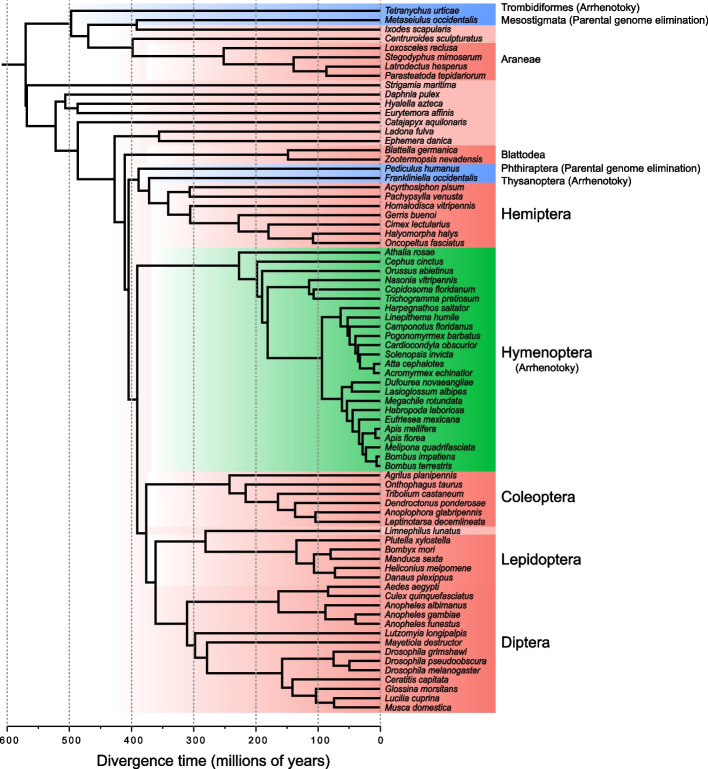
Distribution of haplodiploidy in the phylogeny of 76 arthropod species. The tree is based on the time tree of Thomas et al. [32]. Hymenopterans are colored in green; non-Hymenoptera species are colored in blue; diploid species are in red. Different types of haplodiploidy are in parenthesis. Names of major arthropod orders with more than one species in the tree are next to the corresponding clade

To explore the evolution of mitochondrion-related genes, we collected the genomes of 76 arthropod species with haplodiploid and diploid sex determination systems. We assigned species into three categories, Hymenoptera species (including 24 species from 1 order), non-Hymenoptera haplodiploid species (denoted as “other haplodiploid,” including 4 species from 4 orders), and diploid species (denoted as “diploid,” including 48 species from 12 orders) (Fig. 1 and Additional file 1: Table S1). Nuclear genes and their corresponding orthologous groups were retrieved from a previous study [32]. Mitochondrial genes were downloaded from the National Center for Biotechnology Information (NCBI) if available. For the species without published mitochondrial genomes, we assembled and annotated mitochondrial genomes from whole genome shotgun sequencing data (Additional file 1: Table S1).

In total, we included 38,195 ortholog groups of nuclear genes and 13 mitochondrial genes. Among these orthologous groups, we focused on subsets pertaining to mitochondrial and nuclear interactions, including 13 mitochondrion-encoded OXPHOS genes (mtOXPHOS), 65 nuclear-encoded OXPHOS genes (nucOXPHOS), and 74 nuclear-encoded mitochondrial ribosomal proteins (nucMTRP). As a control, we estimated the evolutionary rates of genes not related to mitochondrion in the genomes, including 77 nuclear-encoded cytosolic ribosomal proteins (nucCRP), 150 nuclear-encoded single-copy genes (nucControlSingle), and 5746 nuclear-encoded genes that were found in ≥ 80% of the species (i.e., ≥ 61 species) and were not necessarily single copy (nucControl) (Table 1, Additional file 1: Table S2).

**Table 1:** Gene categories, functions, and the number of genes within each gene category in the present study

Gene category	Function	Number of orthologous groups
mtOXPHOS	Mitochondrial-encoded OXPHOS protein coding genes	13
nucOXPHOS	Nuclear-encoded OXPHOS protein coding genes	65
nucMTRP	Nuclear-encoded mitochondrial ribosomal protein genes	74
nucCRP	Nuclear-encoded cytosolic ribosomal protein genes	77
nucControlSingle	Single-copy nuclear-encoded genes found in all 76 arthropod species	150
nucControl	Nuclear-encoded genes found in at least 61 arthropod species	5746

### Haplodiploid species have fast-evolving mitochondrion-related genes

**Fig. 2:**
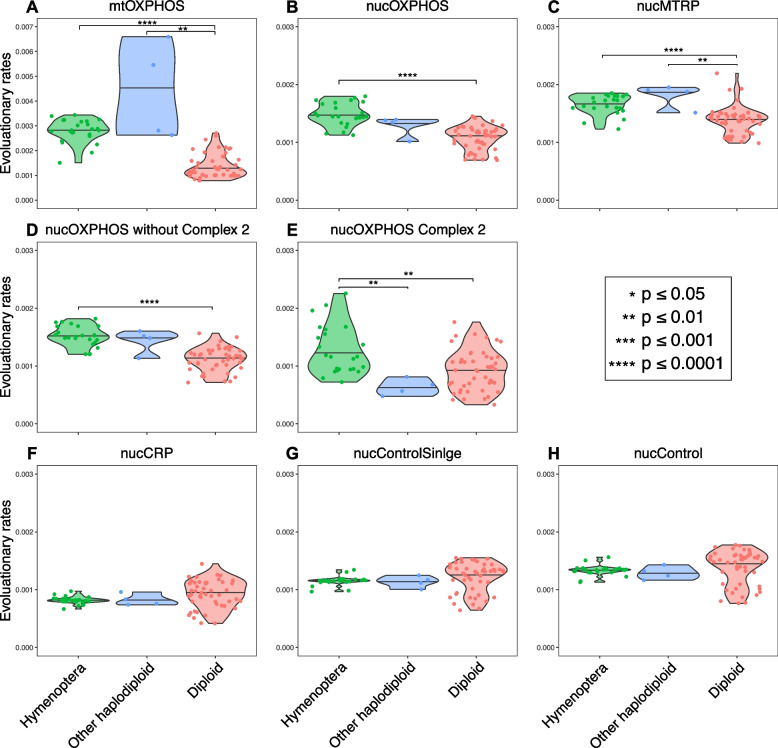
Root-to-tip evolutionary rates (AA/site/MY) of different gene categories among arthropod groups. Asterisks indicate significant differences among hymenopterans (green), other non-Hymenoptera haplodiploid species (blue), or diploid species (red). Note that the y-axis of mtOXPHOS genes is different from nuclear genes due to the elevated evolutionary rates of mitochondrial genes

We first measured the evolutionary rate by concatenating the amino acid sequences of all the genes in the same gene category for each species. We then measured the number of amino acid substitutions per site per million years (AA/site/MY) based on terminal branch length and root-to-tip branch length. In general, terminal branch evolutionary rate and root-to-tip evolutionary rate showed a similar pattern (Fig. 2 and Additional file 2: Fig. S1), except that Hymenoptera nucControl genes showed significantly higher evolutionary rates than diploid species based on terminal branch length. Estimating evolutionary rates using terminal branches is often affected by the divergence time of the branches. Sequences from early diverging lineages could be saturated, leading to an underestimation of the evolutionary rate [40, 41]. At the same time, when lineages are under-sampled, polymorphisms could be treated as recent substitutions leading to the overestimation of evolutionary rates of recent terminal branches [42]. Therefore, more recent terminal branches could have higher evolutionary rates than early diverging branches due to estimation bias. As hymenopterans were well sampled in our study, they often had short divergence times, potentially leading to the observed pattern of fast evolution in their nucControl genes (Additional file 2: Fig. S1). Consistent with this hypothesis, we observed a negative correlation between divergence time and terminal branch evolutionary rate (Additional file 2: Fig. S2). To account for this bias, we estimated the evolutionary rate from the root to each tip in the tree, even though they are not independent of each other.

We first examined the evolutionary rates among different gene groups. Across all species, mtOXPHOS genes (median = 0.00176 AA/site/MY) had the highest evolutionary rates of all gene categories (Additional file 2: Fig. S3). Among nuclear genes, nucCRP (median = 0.00084 AA/site/MY) and nucControlSingle (median = 0.00118 AA/site/MY) were the most conservative gene groups with the lowest evolutionary rate. nucOXPHOS (median = 0.00120 AA/site/MY), nucMTRP (median = 0.00147 AA/site/MY), and nucControl genes showed an intermediate evolutionary rate (median = 0.00136 AA/site/MY). Based on the Kruskal–Wallis test and multiple comparison tests after Kruskal–Wallis, mtOXPHOS and nucMTRP genes evolved significantly faster than nucCRP genes across all species. mtOXPHOS genes in hymenopterans showed a significantly faster evolutionary rate than nucControlSingle and nucControl genes but the pattern was not found in diploid species.

Among different groups of arthropods, mtOXPHOS, nucOXPHOS, and nucMTRP genes evolved significantly faster in hymenopterans than in diploid species, while only mtOXPHOS genes evolved significantly faster in non-Hymenoptera haplodiploid species than diploid species (Fig. 1). As only four non-Hymenoptera species were included in our analysis, the small sample size limited our power to distinguish evolutionary rate differences for the genes. Hymenopterans had fast-evolving mtOXPHOS (median = 0.00282 AA/site/MY), nucOXPHOS (median = 0.00145 AA/site/MY), and nucMTRP (median = 0.00171 AA/site/MY) genes (Fig. 2). Other haplodiploids, including both PGE and arrhenotoky lineages, had fast-evolving mtOXPHOS (median = 0.00413 AA/site/MY) and nucMTRP (median = 0.00189 AA/site/MY) genes. Although the comparison was not significant, other haplodiploid species had higher evolutionary rates (median = 0.00136 AA/site/MY) than diploid species (median = 0.00112 AA/site/MY).

### Coevolution between mitochondrial and nuclear genes

**Fig. 3:**
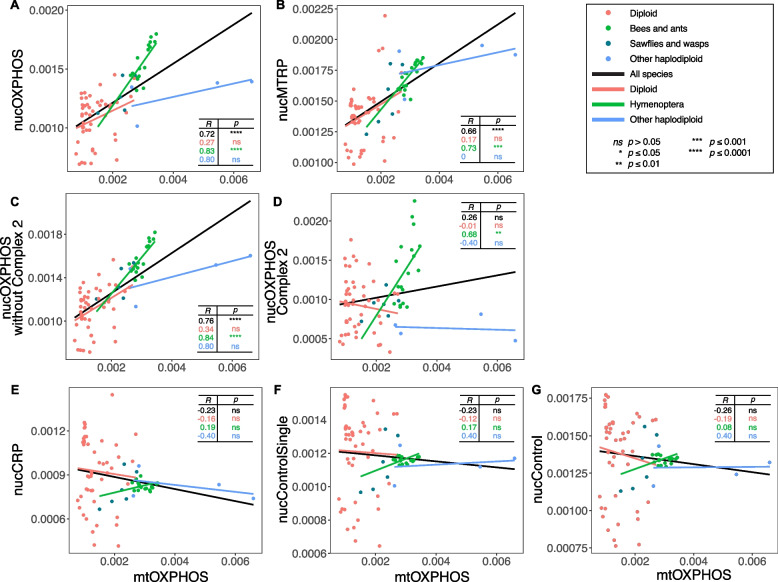
Spearman’s rank correlation of evolutionary rates (AA/site/MY) between mtOXPHOS genes and **A** nucOXPHOS, **B** nucMTRP, **E** nucCRP, **F** nucControlSingle, and **G** nucControl genes based on root-to-tip evolutionary rate. In addition, we also estimated the correlation of evolutionary rates between mtOXPHOS genes and **C** nucOXPHOS genes not from complex 2 and **D** genes only from complex 2. Spearman’s correlation coefficient (*R*) and *p* values (*p*) are used to estimate the correlation between evolutionary rates of mtOXPHOS genes and evolutionary rates of nuclear-encoded gene categories based on all four arthropod groups (black), diploid species (red), hymenopterans (bees and ants: green, sawflies and wasps: dark green), and other non-Hymenoptera haplodiploid species (blue). Linear regression represents the general trend of the correlation

mtOXPHOS and their nuclear counterparts often coevolve to maintain the function of the OXPHOS pathway and mitochondrial replication, transcription, and translation [19, 43]. To investigate the relationships between mtOXPHOS genes and related nuclear genes, we estimated Spearman’s rank correlation coefficients of evolutionary rates between different gene categories. Across all 76 arthropod species, the evolutionary rate of mtOXPHOS genes was significantly correlated with the rates of nucOXPHOS and nucMTRP genes but not with other nuclear genes (Fig. 3 and Additional file 1: Table S3).

When only considering diploid or non-Hymenoptera haplodiploid species, the evolutionary rates of mtOXPHOS and nucOXPHOS genes did not show a strong correlation (Fig. 3A). For example, there was variation among the non-Hymenoptera haplodiploid species: the two PGE haplodiploid species (*M. occidentalis* and *P. humanus*) showed an evolutionary rate of 0.0032 AA/site/MY and 0.0070 AA/site/MY, and the two arrhenotoky species (*T. urticae* and *F. occidentalis*) had an evolutionary rate of 0.0074 AA/site/MY and 0.0029 AA/site/MY, respectively, while their nuclear genes had similar evolutionary rates (Fig. 3B). On the other hand, when only considering hymenopterans, there was a significant correlation, possibly because our study included a more comprehensive selection of species from Hymenoptera than for any other arthropod orders.

It has been hypothesized that nucOXPHOS genes that do not directly interact with mitochondrial genes tend to have less correlated evolutionary rates with mitochondrial genes [19, 44]. We found that mtOXPHOS genes and nucOXPHOS genes excluding OXPHOS complex 2 (Fig. 3C, Additional file 1: Table S3) have a better correlation than that including OXPHOS complex 2 (Fig. 3A) in arthropods. Arthropod mtOXPHOS genes and nucOXPHOS complex 2 genes have no significant correlation (Fig. 3D). Only hymenopterans show a strong correlation between mtOXPHOS genes and nucOXPHOS complex 2 genes. This observed correlation in hymenopterans is likely due to the evolutionary rate variations within hymenopterans that bees and ants tend to have higher evolutionary rates than sawflies and wasps in their nucOXPHOS genes (Fig. 3A).

### Bees and ants have elevated mitochondrion-related nuclear gene evolutionary rates

**Fig. 4:**
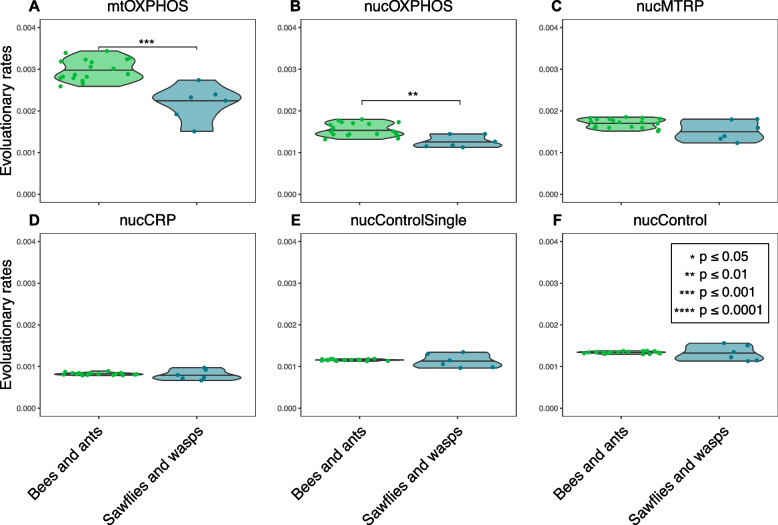
Root-to-tip evolutionary rates of different gene categories among hymenopterans. Asterisks indicate significant differences between bees and ants (green) and sawflies and wasps (dark green)

To investigate rate variation among hymenopterans, we assigned hymenopterans to two groups: eusocial Hymenoptera (including bees [superfamily: Apoidea] and ants [superfamily: Formicoidea; family: Formicidae]) and basal lineages (including sawflies [superfamilies: Cephoidea and Tenthredinoidea] and wasps [superfamilies: Chalcidoidea and Orussoidea]). As sawflies and wasps have higher divergence times than bees and wasps, we used tip-to-root evolutionary rates for this analysis. We found that bees and ants had significantly higher evolutionary rates in mtOXPHOS and nucOXPHOS genes than sawflies and wasps (Fig. 4).

To confirm our findings based on root-to-tip evolutionary rates and to consider the unbalanced sampling of bees, ants, wasps, and sawflies, we also estimated the terminal branch evolutionary rate by subsampling within hymenopterans. For each subsampling, we sampled one hymenopteran without replacement with the rest of the arthropods to keep the divergence time consistent among subsamples. Subsampling results were consistent with root-to-tip estimations that bees and ants had significantly higher evolutionary rates in mtOXPHOS and nucOXPHOS genes than sawflies and wasps (Additional file 2: Fig. S4).

### Gene family expansion and contraction of haplodiploid and diploid arthropods

Lastly, as complete genomes were used, we investigated gene family expansions and contractions related to haplodiploidy. Based on a feature selection analysis using the R package, Boruta [45], multiple mitochondrion-related genes had significantly different copy numbers among haplodiploid and diploid species. For example, 3 out of 35 genes had significantly different copy numbers between haplodiploid/diploid species (Additional file 1: Table S4). Between bees/ants and sawflies/wasps, 9 out of 26 genes with significantly different copy numbers were mitochondrion-related. There were two copies of the mitochondrial translation elongation factor gene (EOG8Z0DM0, FlyBase IDs mEFTu1 [FBpp0086790] and mEFTu2 [FBpp0088054]) in sawflies and wasps but only one copy in most of the bees and ants. Interestingly, different from mEFTu1, mEFTu2 is overexpressed in the testis of *Drosophila melanogaster* [46, 47]. Changes in the gene family could relate to mitochondrial functions in bees and ants.

## Discussion

The coevolution of mitochondrial and nuclear genes is critical to mitochondrial functions. The mitochondrion is often uniparentally inherited and effectively haploid. As a result, the mitochondrion has a reduced effective population size relative to diploid nuclear genes and is prone to accumulation of deleterious mutations and, therefore, evolves faster than nuclear genes. The rate of molecular evolution in the mitochondrial genome varies dramatically among arthropods; however, the reasons for the accelerated mitochondrial rate in some arthropods remain unknown. Previous studies hypothesized that the elevated evolutionary rates in mitochondrial genomes of hymenopterans are due to their small effective population size from haplodiploidy, parasitism, or selection in the mitochondrion [28, 48]. To test these hypotheses, we investigated the evolutionary rate of mitochondrial and nuclear genes across five haplodiploid lineages, including three haplodiploid lineages in Insecta and two haplodiploid lineages in Acari (Fig. 1). These five haplodiploid lineages have arisen independently, providing a unique opportunity to comprehensively explore the evolution of mitochondrion-related genes.

### Small population size is not the reason for fast-evolving mitochondrion-related genes in haplodiploid species

We showed that all haplodiploid species tested here, across 500 million years of evolution, have fast-evolving mitochondrial genes and mitochondrion-related nuclear genes (Fig. 2). Our observation suggests that haplodiploidy is linked to fast-evolving mitochondrial genes. Our results are consistent with other studies showing elevated evolutionary rates in mitochondrial genes of hymenopterans [19–21] and *P. humanus* [49].

Elevated evolutionary rates also correlate with fast mitochondrial gene rearrangement rates in arthropods [50, 51]. Previous studies have shown that most of the haplodiploid species included in this study have elevated mitochondrial rearrangement rates, including species in Hymenoptera [52], Thysanoptera (including *F. occidentalis*) [53, 54], and Phytoseiidae (including *M. occidentalis*) [55–57]. The most extreme mitochondrial rearrangement case is in *Pediculus* species, where instead of a single circular chromosome, the mitochondrial genome fractured into 20 circular mini-chromosomes [58]. Besides *Pediculus*, fragmented mitochondria and endosymbionts have arisen in multiple lineages, such as mini-chromosomes in *Silene* [59] and nematodes [60, 61], and fragmented genomes of endosymbiont *Hodgkinia* in cicadas [62], which all have elevated evolutionary rates. The fragmented genomes of mitochondria and symbionts are possibly nonadaptive for their hosts [63, 64].

For nuclear genes, our finding that mitochondrion-related genes (nucOXPHOS and nucMTRP) have fast evolutionary rates but that other nuclear genes (nucControlSingle and nucControl) do not (Figs. 2 and 3) rejects the hypothesis that the reduced effective population size caused by haplodiploidy or parasitism is the reason for the fast-evolving mitochondrial genomes. As both haplodiploidy and parasitism lead to a small population size, all nuclear genes should exhibit rapid evolution. Even though we included a larger set of nucControl genes than previous studies, we still did not find a genome-wide pattern of rapid evolution.

### The nuclear compensation hypothesis could explain the fast-evolving mitochondrial-related genes in haplodiploid species

Alternatively, both nuclear compensation and selection on the OXPHOS pathway hypotheses could lead to the observed pattern. The nuclear compensation explanation is based on the observation that the mitochondrion often has fast evolutionary rates and accumulates more deleterious mutations than nuclear genes [5, 8], and thus, nuclear genes are hypothesized to be under positive selection to compensate for the deleterious changes in the mitochondrion [27]. In this scenario, mitochondrion-related nuclear genes are under positive selection but not the mitochondrial genes and unrelated nuclear genes. Non-Hymenoptera haplodiploid species show this pattern of rapid evolution in the nucOXPHOS complexes that consist of both mito-nuclear genes but not in the complexes with only nuclear-encoded genes (complex 2) (Fig. 2E). The observed evolutionary rate differences for OXPHOS genes are consistent with the nuclear compensation explanation. For haplodiploid species, haploid males express recessive mutations could lead to more efficient selection to purge deleterious and fix beneficial mutations, expediting the nuclear compensation process.

We were not able to reliably calculate *dN/dS* ratio (the ratio of the number of non-synonymous nucleotide substitutions per non-synonymous site to the number of synonymous nucleotide substitutions per synonymous site) for haplodiploid species, due to the deep divergence among the 76 arthropods. Our previous study, based on a comprehensive sampling of species in Hymenoptera, Coleoptera, Lepidoptera, and Diptera, demonstrated that 17 out of 23 tested nucOXPHOS genes of hymenopterans had an elevated *dN/dS* ratio compared to other holometabolous species, but only 2 out of 13 mitochondrial genes of hymenopterans had an elevated *dN/dS* ratio compared to other holometabolous species [21]. As nucOXPHOS genes under positive selection are important genes for mitochondrial functions, it is likely that the elevated *dN/dS* ratio is due to positive selection to compensate for the deleterious mutations in fast-evolving mitochondrial genes. In another arthropod example, positive selection was detected in nucOXPHOS and nucMTRP but not mitochondrial genes for copepod species with fast-evolving mitochondrial genes [65, 66]. Therefore, it is possible that the fast-evolving nucOXPHOS genes found in haplodiploid species were due to compensation for the fast-evolving mtOXPHOS genes.

### The positive selection hypothesis could explain the fast-evolving mitochondrion-related genes in bees and ants

The positive selection on OXPHOS genes hypothesis claims that both mitochondrial and nuclear genes are under positive selection. Although most studies [21, 43, 67] have shown that mitochondrial genes often have a low *dN/dS* ratio supporting the nuclear compensation hypothesis, Piccinini et al. [44] found that mtOXPHOS genes of bivalves had similar *dN/dS* ratios as nucOXPHOS genes, suggesting positive selection on the whole OXPHOS pathway, possibility due to adaptation to environmental stress.

Bees and ants experience different evolutionary patterns than other haplodiploid species. None of the haplodiploid species except bees and ants showed a correlation between mtOXPHOS genes and nucOXPHOS complex 2 genes (Fig. 3D), although genes in complex 2 do not directly interact with mtOXPHOS genes. It is possible that the whole OXPHOS pathway of bees and ants is under positive selection due to their high metabolic demand. For example, bees and ants often maintain their nest temperature using metabolic heat [68, 69]. Bees and ants are also central place foragers. A recent study has found that the OXPHOS genes of pollinating fig wasps (superfamily: Chalcidoidea) evolved significantly faster than those genes of non-pollinating fig wasps [70]. Five of the OXPHOS genes in pollinators showed signals of positive selection possibly due to energetic demand during pollination [70].

In addition, we propose another explanation for the elevated mitochondrial gene evolutionary rate that haplodiploid species could accumulate deleterious mutations on the mitochondrial recombination, replication, and repair genes due to small population size. The disruption of mitochondrial recombination, replication, or repair genes (RRR genes) could cause increased substitution rates in mitochondrial genomes. Such cases have been found in *D. melanogaster* [71], *Arabidopsis thaliana* [72], and African killifishes [73]. The observed elevated evolutionary rates on nucOXPHOS complex 2 of bees and ants (Figs. 3 and 4) also support this explanation, as eusocial hymenopterans have even smaller population sizes than sawflies and wasps [25, 74] potentially leading to a higher chance of deleterious mutations occurring on mitochondrion-related genes. Recent studies [75, 76] also found that low copy numbers of mitochondrial DNA could lead to high mitochondrial evolutionary rates due to the less efficient homologous recombination for mitochondrial DNA repair. The nuclear compensation, positive selection, and less efficient mitochondrial repair system are not exclusive to each other and could work together affecting the mito-nuclear evolution of bees and ants.

### Gene family expansions and contractions in different haplodiploid groups

Besides evolutionary rates, we also investigated gene gains and losses in haplodiploid species. Overall, arthropods have conserved copy number for mitochondrion-related genes due to the functional importance of mitochondrion. Only a few mitochondrion-related gene families were found to have significant expansions or contractions in haplodiploid species. We also found several gene losses with mitochondrion-related GO terms, which could affect the function of mitochondrial genes. Many bees and ants have lost one of the mitochondrial translation elongation factor gene copies, *mEFTu2*. The gains and losses of mitochondrion-related genes could affect mitochondrion evolution. For example, the loss of the mitochondrial single-stranded binding protein has been associated with the mitochondrial mini-chromosomes in the *P. humanus* genome [77].

### Biased sampling efforts of genomes could affect the estimation of evolutionary rates

One caveat to our study is the biased genome sequencing efforts devoted to hymenopterans due to their agricultural importance [78, 79]. Of the 76 studied arthropod species, 26 were hymenopterans, and only four species represented four non-Hymenoptera haplodiploid orders. This biased sampling effort limited our interpretation of the global effects of haplodiploidy on evolutionary rates. Although we performed subsampling and simulation to take this sampling bias into account, more genomes from other haplodiploid orders could vastly improve our understanding of haplodiploidy and its effects on genome evolution in the future.

## Conclusions

We studied the evolutionary and co-evolutionary dynamics of mitochondrial and nuclear genes across major haplodiploid arthropod lineages. We found elevated mitochondrion-related gene evolution in all five haplodiploid lineages. Our findings suggest that the small population size of haplodiploid species is not likely to be the reason for the fast-evolving mitochondrial genes. But investing different gene groups, we propose three alternative explanations, including nuclear compensation, positive selection on the OXPHOS pathway, and deleterious substitutions on the mitochondrial RRR genes. We also showed several gene family gains and losses in haplodiploid species, suggesting the unique evolution history of their mitochondrion-related genes.

## Methods

### Taxon sampling and sequence acquisition

The genome sequences of 28 arthropod species from the Arthropod 5000 Genomes Initiative (i5K) project [30] and 48 previously sequenced arthropods spanning 21 arthropod orders were used in this study (Additional file 1: Table S1). Within the 76 species, 28 species were haplodiploid, including 24 species from Hymenoptera and 4 species from Trombidiformes, Mesostigmata, Phthiraptera, and Thysanoptera (Fig. 1).

A set of 38,195 ortholog groups was obtained from Thomas et al. [32] based on the orthologous gene prediction of OrthoDB8 [80]. Two categories of nuclear genes related to mitochondrial function were used in our study: (i) nuclear-encoded OXPHOS genes (nucOXPHOS) and (ii) nuclear-encoded mitochondrial ribosomal protein genes (nucMTRP) [19, 81]. To estimate the background evolutionary rate of genes in the nuclear genome, three gene categories with no relationship to mitochondrial genes were used, including (i) nuclear-encoded cytosolic rRNA protein genes (nucCRP), (ii) 150 single copy nuclear-encoded genes (nucControlSingle) (Thomas et al. [32]), and (iii) all the nuclear-encoded genes (nucControl).

Multiple approaches were used to extract groups of orthologous genes in the different categories described above from the complete set of orthologs. For nucOXPHOS genes, the OXPHOS gene annotations of *Drosophila melanogaster* [82, 83] were used to search the *D. melanogaster* gene identifiers (IDs) in each ortholog group (Additional file 1: Table S2). For nucMTRP genes, 75 genes under the FlyBase ID “FBgg0000059” were used to search the *Drosophila melanogaster* gene IDs in ortholog groups. For nucCRP genes, 93 genes under the FlyBase ID “FBgg0000141” were used to match the genes in ortholog groups [19, 81]. Of the 38,195 ortholog groups, 150 ortholog groups had only 1 copy in any of the species. These 150 ortholog groups were used as nucControlSingle genes. As RAxML [84] treated gaps as undetermined base pairs, missing genes could inflate the evolutionary rate estimation. Therefore, 5746 ortholog groups that could be found in ≥ 80% of the species (at least 61 species) were used as background genes (nucControl gene category).

### Assembly and annotation of mitochondrial genomes

Reference mitochondrial genomes of 32 arthropod species are available on NCBI. Mitochondrial genes of 12 species have already been annotated from transcriptome data in our previous study (Li et al. [21]). For the rest of the species, we first assembled the mitochondrial genome from the available genome sequence data using MITObim version 1.9 [85]. To minimize the computational requirements, we extracted between 12,500,000 and 100,000,000 reads for mitochondrial genome assembly (Additional file 1: Table S1). If a mitochondrial reference genome from the same genus was available, “-quick” option of MITObim was used where mitochondrial genomes were assembled based on the reads with a certain kmer overlap to the mitochondrial reference. If a mitochondrial genome from the same genus was not available, the COI barcode of the same species was used as a seed for assembly. Assemblies from MITObim were annotated online using MITOS version 2 [86] based on NCBI RefSeq 81 and the invertebrate genetic code. We were able to assemble and annotate 23 arthropod species using this approach. For the remaining 9 species without mitochondrial genomes and their mitochondrial genomes could not be assembled from the genomic data, a reference mitochondrial genome from a closely related species was used in the analysis (Additional file 1: Table S1). The protein-coding genes of the mitochondrial genomes were used for subsequent mitochondrial OXPHOS gene (mtOXPHOS) evolutionary rate analysis.

### Estimating evolutionary rates

Due to the deep divergence, we used amino acid sequences of orthologous groups to estimate evolutionary rates. In cases where a species had multiple paralogs within an orthologous group, a random paralog was selected for subsequent analysis. The amino acid sequences of each orthologous group were first aligned using the L-INS-i algorithm in MAFFT version 7.475 [87]. GBlocks version 0.91b [88] was then used to remove the low-quality regions of the alignment with “-t = p -b4 = 5 -b5 = a -e = -gb” meaning the input aligned sequences were amino acid alignment, the minimum length of a block was 6 amino acids, and gaps were allowed. Lastly, genes in the same gene category (i.e., mtOXPHOS, nucOXPHOS, nucMTRP, nucCRP, nucControlSingle, and nucControl) were concatenated.

To estimate evolutionary rates, branch lengths of gene trees were estimated using the concatenated amino acid sequence alignments with RAxML version 8.2.3 and the same fixed topology from Thomas et al. [32]. RAxML version 8.2.3 [84] was used with the PROTGAMMAAUTO model option, which automatically selected the best-fitting amino acid substitution model based on the log-likelihood value and approximated across-site rate heterogeneity with a gamma distribution. In RAxML, the “-t” option was used to estimate branch lengths based on the given topology. We estimated both the terminal branch length and the root-to-tip branch length, which included the terminal branch length and all the internal nodes leading to the common ancestor of arthropods, using phytools version 0.6–44 [89]. We calculated the evolutionary rate as the branch length estimation divided by the divergence time (MYA). The divergence time of each species was retrieved from the time tree of Thomas et al. [32].

To evaluate whether the gene family size of control genes (5746 genes) biased the estimation of evolutionary rate, 1000 random subsamplings were performed based on the gene number and gene family size of nucOXPHOS and nucMTRP genes. The evolutionary rate was estimated in the same way above. Spearman’s rank correlation coefficient (cor.test function in R) was used to estimate the differences between the evolutionary rate of subsamplings and 5746 control genes.

### Statistical analysis of gene evolutionary rates

R version 3.4.4 [90] was used to perform statistical tests. Kruskal–Wallis test (kruskal.test) and multiple comparison tests after Kruskal–Wallis (kruskalmc) from pgirmess package version 2.0.3 [91] were used to test the significance of gene evolutionary rate differences between Hymenoptera species, other non-Hymenoptera haplodiploid species, and diploid species. Spearman’s rank correlation coefficient (cor.test function in R) was used to calculate the correlation of rates between gene groups. The PCA package in R was used to find the principal components that explain the pattern of evolutionary rates within arthropod species. ggplot2 version 2.2.1.9 [92] and ggtree version 1.10.5 [93] were used to visualize data and phylogenetic trees.

### Mitochondrion-related gene family expansion and contraction in arthropods

Gene family expansions and contractions were estimated based on the number of genes in each ortholog group and the time tree using Dupliphy version 1.0 [94]. To explore the unique gene family expansions and contractions in haplodiploid species and Hymenoptera species, a random forest algorithm was used to select significant features using the R package Boruta version 5.3.0 [45]. Three different sets of species were used for feature selection analysis: (a) haplodiploid species versus diploid species, (b) hymenopterans versus diploid species, and (c) haplodiploid species except hymenopterans versus diploid species. Gene copy numbers were used as input attributes to separate the two groups. For gene families that separate different groups of insects based on the Boruta approach, we used a rough fix of tentative attributes (TentativeRoughFix) to judge whether these gene families were significantly important.

## Supplementary Information

Additional file 1: Tables S1–S4. Table S1 Arthropod species, genomes, and mitochondrial genomes used in the study. Table S2 Nuclear-encoded OXPHOS, mitochondrial ribosomal protein genes, and cytosolic ribosomal proteins used in the study. Table S3 Spearman’s rank correlation analysis of root-to-tip evolutionary rates between mitochondrial genes and other gene categories. Table S4 Genes that separate different arthropod groups

Additional file 2: Figures S1–S4. Fig. S1 Terminal branch evolutionary ratesof different gene categories among arthropod groups. Asterisks indicate significant differences among hymenopterans, other non-Hymenoptera haplodiploid species, or diploid species. Fig. S2 Correlation between divergence time and terminal branch evolutionary ratesbased on Spearman’s rank correlation. Spearman’s rank correlation coefficient is estimated based on all species from hymenopterans, other non-Hymenoptera haplodiploid species, or diploid species. Fig. S3 Root-to-tip evolutionary rate of different gene categories among all species, hymenopterans, other haplodiploid species, and diploid species. Significance was denoted based on letters. Letters on top of the violin plots denote significant differences based on the Kruskal-Wallis test and multiple comparison tests after Kruskal-Wallis. Letters were ordered alphabetically based on the median of the distributions from the highest to the lowest. Fig. S4 Terminal branch evolutionary rate of different gene categories among hymenopterans with subsampling strategy. In each subsampling, one hymenopteran with the rest of the arthropods to keep the divergence time consistent among subsamples

## Data Availability

The amino acid sequences of all the gene categories and scripts used in the current study are available at https://github.com/lyy005/Mitochondrial_nuclear_gene_evolution_in_arthropods (last accessed Sept 26, 2024). The information of the 76 arthropod species used in this study is available at https://arthrofam.org. Complete mitochondrial genomes assembled in this study are under the NCBI accession numbers: MN842135–MN842145.
