# Supplementary material for: Rapid evolution of mitochondrion-related genes in haplodiploid arthropods
Source: BMC Biol. 2024 Oct 10;22:229. doi: 10.1186/s12915-024-02027-4 (PMC11465517; doi:10.1186/s12915-024-02027-4)
Supplement: Supplementary file 2 — Additional file 2: Figures S1–S4. Fig. S1 Terminal branch evolutionary ratesof different gene categories among arthropod groups. Asterisks indicate significant differences among hymenopterans, other non-Hymenoptera haplodiploid species, or diploid species. Fig. S2 Correlation between divergence time and terminal branch evolutionary ratesbased on Spearman’s rank correlation. Spearman’s rank correlation coefficient is estimated based on all species from hymenopterans, other non-Hymenoptera haplodiploid species, or diploid species. Fig. S3 Root-to-tip evolutionary rate of different gene categories among all species, hymenopterans, other haplodiploid species, and diploid species. Significance was denoted based on letters. Letters on top of the violin plots denote significant differences based on the Kruskal-Wallis test and multiple comparison tests after Kruskal-Wallis. Letters were ordered alphabetically based on the median of the distributions from the highest to the lowest. Fig. S4 Terminal branch evolutionary rate of different gene categories among hymenopterans with subsampling strategy. In each subsampling, one hymenopteran with the rest of the arthropods to keep the divergence time consistent among subsamples [file 12915_2024_2027_MOESM2_ESM.zip › FigureS3_root2tip.ai5.pdf]

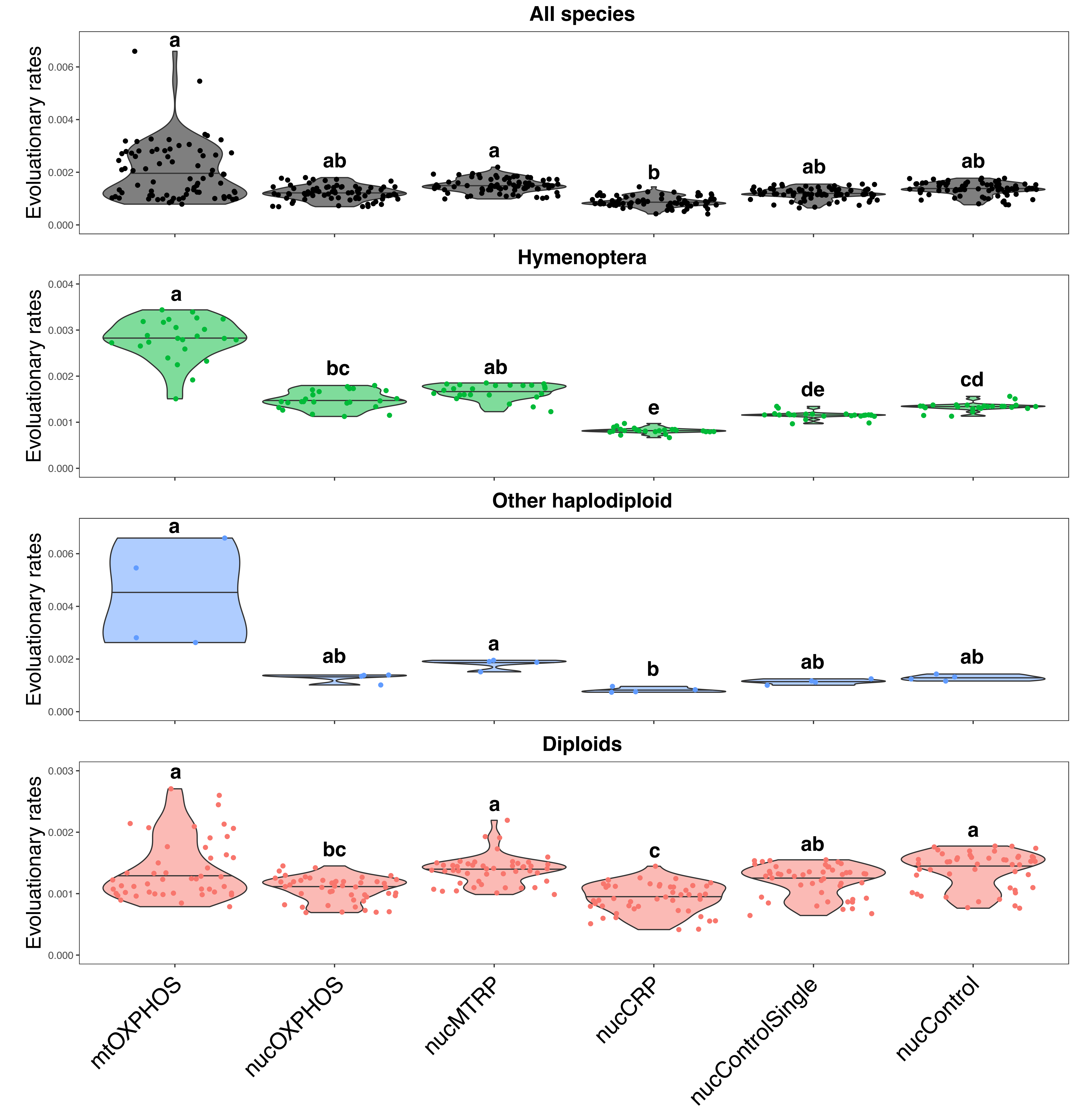

All species
Hymenoptera
Other haplodiploid
Diploids
Evoluationary rates
0.000
0.001
0.002
0.003
0.004
0.006
a
ab
b
bc
c
cd
de
e
mtOXPHOS
nucOXPHOS
nucMTRP
nucCRP
nucControlSingle
nucControl
